# Genomic Prediction with Pedigree and Genotype × Environment Interaction in Spring Wheat Grown in South and West Asia, North Africa, and Mexico

**DOI:** 10.1534/g3.116.036251

**Published:** 2016-11-30

**Authors:** Sivakumar Sukumaran, Jose Crossa, Diego Jarquin, Marta Lopes, Matthew P. Reynolds

**Affiliations:** * Global Wheat Program, International Maize and Wheat Improvement Center (CIMMYT), El Batan, Texcoco CP 56237, Mexico; † Biometrics and Statistics Unit, CIMMYT, El Batan, Texcoco CP 56237, Mexico; ‡ Department of Agronomy and Horticulture, University of Nebraska, Lincoln, Nebraska 68583; § Global Wheat Program, CIMMYT, Emex, 06511 Ankara, Turkey

**Keywords:** genomic prediction, pedigree-based prediction, WAMI, spring wheat, GBLUP, genomic selection, GenPred, Shared Data Resources

## Abstract

Developing genomic selection (GS) models is an important step in applying GS to accelerate the rate of genetic gain in grain yield in plant breeding. In this study, seven genomic prediction models under two cross-validation (CV) scenarios were tested on 287 advanced elite spring wheat lines phenotyped for grain yield (GY), thousand-grain weight (GW), grain number (GN), and thermal time for flowering (TTF) in 18 international environments (year-location combinations) in major wheat-producing countries in 2010 and 2011. Prediction models with genomic and pedigree information included main effects and interaction with environments. Two random CV schemes were applied to predict a subset of lines that were not observed in any of the 18 environments (CV1), and a subset of lines that were not observed in a set of the environments, but were observed in other environments (CV2). Genomic prediction models, including genotype × environment (G×E) interaction, had the highest average prediction ability under the CV1 scenario for GY (0.31), GN (0.32), GW (0.45), and TTF (0.27). For CV2, the average prediction ability of the model including the interaction terms was generally high for GY (0.38), GN (0.43), GW (0.63), and TTF (0.53). Wheat lines in site-year combinations in Mexico and India had relatively high prediction ability for GY and GW. Results indicated that prediction ability of lines not observed in certain environments could be relatively high for genomic selection when predicting G×E interaction in multi-environment trials.

Wheat is the most widely cultivated cereal crop in the world, and provides 20% of the protein and calories consumed by the world population (FAOSTAT). Several studies have reported that the present rate of genetic gain in spring wheat is <1% yr^−1^ (Aisawi *et al.* 2015; Sayre *et al.* 1997; Manes *et al.* 2012; Lopes *et al.* 2012); that rate needs to improve to meet future wheat demand (Reynolds *et al.* 2012). This can be done through improvements in plant structure and reproduction, and in crop physiology (radiation use efficiency), as well as improved genotyping or phenotyping methods, increased genetic diversity of breeding germplasm, or through the use of complementary genomic selection approaches in plant breeding (Reynolds *et al.* 2009; Tester and Langridge 2010).

Traditional breeders use the pedigree selection method for breeding most crops, which requires several generations of testing and advancing the lines. An alternative method is marker-assisted selection (MAS), where markers associated with genes of major effect are used (Spindel *et al.* 2015). The first to propose predicting breeding values of complex traits for unobserved phenotypes using all available high density markers were Meuwissen *et al.* (2001). This initial study was followed, in plants, by Bernardo and Yu (2007), who demonstrated, by simulation, that whole genome regression predicts complex traits more accurately than using only a few markers. These seminal investigations led to the application of different statistical parametric and nonparametric genomic models with pedigree information in different crops (Crossa *et al.* 2010, 2014; Jarquin *et al.* 2014; Pérez-Rodríguez *et al.* 2015; Velu *et al.* 2016; de los Campos *et al.* 2013; de los Campos and Pérez-Rodríguez 2013; Arruda *et al.* 2015).

All the initial genomic prediction models were developed for single-environment prediction. However, GS can accelerate genetic gains in wheat breeding, especially when multi-environment testing of lines is routine in their development and release (Braun *et al.* 2010). Multi-environment testing is prone to high levels of genotype × environment (G×E) interaction due to varying climatic zones, dynamic weather parameters, and different management factors. Burgueño *et al.* (2012) were the first to use marker- and pedigree-based Best Linear Unbiased Predictor (BLUP) models for assessing G×E under genomic prediction; these models account for correlated environmental structures, and thus predict performance of unobserved phenotypes in several environments. Heslot *et al.* (2014) incorporated crop modeling data for studying genomic G×E, and Jarquín *et al.* (2014) proposed a random effect genomic BLUP (GBLUP) model, where the main effect and the G×E interaction effects of markers and environmental covariates are introduced via covariance structures of markers and environmental covariables in a reaction norm model.

The reaction norm model of Jarquín *et al.* (2014) has been widely used in multi-environment data of different crops, including wheat data with pedigree and genomic information and their interaction with environments (Pérez-Rodríguez and de los Campos 2014; Crossa *et al.* 2016; Velu *et al.* 2016). This model is flexible and allows the incorporation of highly dimensional environmental covariable data. Furthermore, a similar genomic G×E model was recently developed and used on wheat breeding data, with the novelty that it decomposes the total marker × environment effect into a marker main effect across all the environments, and a marker-specific effect for each environment (López-Cruz *et al.* 2015). A recent study by Crossa *et al.* (2015) showed how the marker × environment model can be used both as a prediction model, by means of shrinkage regression, and/or as a variable selection to estimate marker effects.

Previous genomic and pedigree studies on assessing the prediction ability of G×E have considered a very limited number of environments; they usually included combinations of sites under a few agronomic management systems (*i.e.*, levels of managed drought and heat stress). In the present study, we assess the genomic prediction ability of several models within the framework of the reaction norm model of Jarquín *et al.* (2014) using the Wheat Association Mapping Initiative (WAMI) panel-designed to evaluate G×E for grain yield (GY), while avoiding the confounding effects of extreme phenology (Lopes *et al.* 2015). The main objective of this study was to detect prediction ability of different international sites established in different years, with the objective of examining and identifying possible key testing sites to be further used in a genomic-assisted breeding program. A total of 287 spring wheat lines included in the WAMI data were grown in international multi-environment trials in 18 site-year combinations in South and West Asia, North Africa, and Mexico. Traits included in this study are grain yield (GY), grain number (GN), thousand-grain weight (GW) and thermal time for flowering (TTF).

The WAMI panel used in this study is very appropriate for studying genomic and pedigree prediction because its lines were phenotyped under a very diverse set of environments around the world. Also, the WAMI panel has already been studied for several complex traits: adaptation to plant density (Sukumaran *et al.* 2015b), grain yield and yield components (Sukumaran *et al.* 2015a), drought stress (Edae *et al.* 2014), and earliness *per se* (Sukumaran *et al.* 2016).

## Materials and Methods

### The genetic material

The WAMI population was assembled from the elite advanced wheat nurseries distributed through the International Wheat Improvement Network (IWIN). It consists of 287 diverse elite lines selected from nurseries bred for high yield potential environments (Lopes *et al.* 2015; Sukumaran *et al.* 2015a,b).

### Phenotyping and genotyping

**Figure 1:**
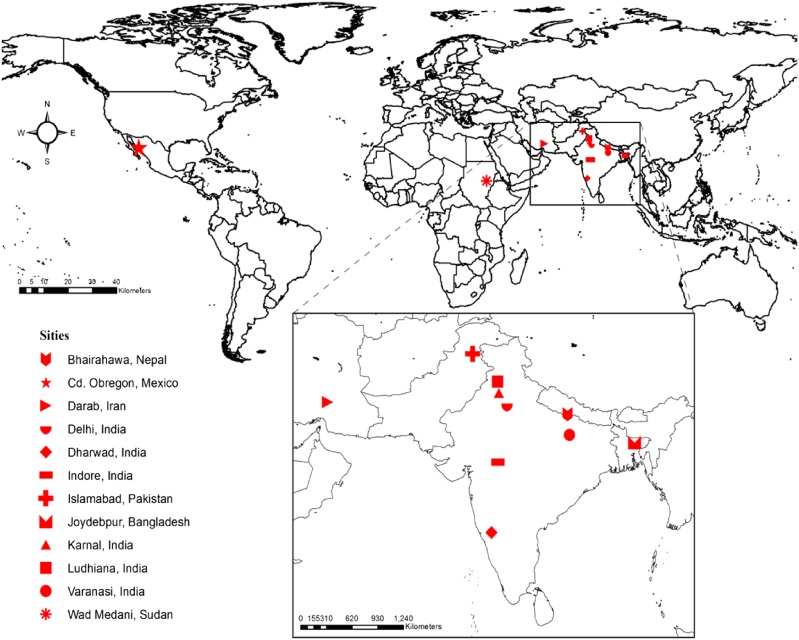
Geographical distribution of the sites where the wheat association mapping initiative (WAMI) panel was grown in 2010–2011 in some of the major wheat growing areas. The map was created using ArcGIS Desktop Arcmap software.

The WAMI population was phenotyped in major wheat-growing areas of India, Pakistan, Nepal, Bangladesh, Iran, Egypt, Sudan, and Mexico. These growing environments are diverse in terms of rainfall, heat stress, drought stress, and solar radiation patterns. Phenotyping was conducted at the following locations: Bangladesh Agricultural Research Institute (BARI), Joydebpur, Bangladesh (BGLD J); Indian Agricultural Research Institute (IARI), Delhi (India D); University of Agricultural Sciences, Dharwad, Karnataka (India H); Indian Institute of Wheat and Barley Research, Karnal, India (India K); Punjab Agricultural University, Ludhiana, India (India L); Darab Hassan Abad, Fars, Iran (Iran D); Banaras Hindu University, Varanasi (India V); National Wheat Research Program, Bhairahawa, Rupandehi (Nepal B); and National Agricultural Research Centre, Islamabad (PAK I). In addition, phenotyping was done under four different treatments at the Norman Borlaug Experiment Station, Cd. Obregon, Sonora, Mexico: irrigated yield potential (Mex I), heat stress (Mex H), drought stress (Mex D), and heat and drought stress (Mex HD) (Figure 1). Table 1 shows the countries, locations, and abbreviations used in this study, as well as the four traits that were recorded and analyzed: GY per square meter, GN per square meter, GW estimated using standard protocols (Sayre *et al.* 1997), and TTF estimated based on a base temperature of zero and the sowing date. Minimum and maximum temperatures, and the coordinates of the environments, were described in an earlier publication (Sukumaran *et al.* 2016).

**Table 1:** Descriptive statistics of the wheat association mapping initiative (WAMI) panel grown in several international environments

Country	Location	Abbreviation	GY (Tons/Ha)	GN (Number)	GW	TTF
Bangladesh	Joydebpur	BGLD J10	2.2 ± 0.3	7314 ± 1249	30.9 ± 4.1	1260 ± 54
		BGLD J11	3.4 ± 0.5	11,326 ± 1807	31.1 ± 4.1	1261 ± 64
India	Delhi	India D10	3.8 ± 0.6	11,520 ± 2018	33.6 ± 2.3	1343 ± 22
	Dharwad	India H10	3.1 ± 0.4	11,415 ± 1914	27.6 ± 3.1	1336 ± 13
	Indore	India I11	5.7 ± 0.9	17,542 ± 3125	32.7 ± 3.7	1304 ± 46
	Karnal	India K10	4.2 ± 0.7	11,729 ± 2185	36.5 ± 4.1	1223 ± 46
	Ludhiana	India L11	4.3 ± 0.7	11,386 ± 1502	38.3 ± 2.8	1422 ± 64
	Varanasi	India V10	3.2 ± 0.6	14,189 ± 3079	23.2 ± 2.3	1242 ± 25
Mexico	Drought*^a^*	Mex D10	3.7 ± 0.4	9902 ± 1204	37.7 ± 4.5	1175 ± 45
	Heat*^a^*	Mex H10	4.0 ± 0.4	13,341 ± 1849	30.5 ± 3.5	976 ± 41
	Heat drought*^a^*	Mex HD10	3.4 ± 0.4	11,831 ± 1931	29.2 ± 4.2	959 ± 32
	Irrigated*^a^*	Mex I10	7.0 ± 0.3	15,032 ± 1201	43.4 ± 4.5	1339 ± 33
Nepal	Bhairahawa	Nepal B10	2.7 ± 0.5	8654 ± 1958	31.9 ± 4.4	1435 ± 45
		Nepal B11	2.5 ± 0.5	7770 ± 1618	32.9 ± 4.3	1377 ± 49
Pakistan	Islamabad	Pak I10	3.2 ± 0.9	10,836 ± 3158	29.8 ± 3.1	1204 ± 74
		Pak I11	6.9 ± 2.0	21,920 ± 6417	31.8 ± 3.0	1105 ± 38
Iran	Darab	Iran D10	5.3 ± 0.9	16,299 ± 2690	33.0 ± 3.3	1343 ± 22
Sudan	Wad Medani	Sudan W10	2.9 ± 0.4	8534 ± 1664	34.7 ± 3.9	1474 ± 99

a Campo Experimental Norman E. Borlaug (CENEB), Mexico, different environments.

The WAMI panel was genotyped using 90K Illumina SNPs array (Sukumaran *et al.* 2015a). From the polymorphic SNPs after using a minor allele frequency cut-off of 5%, 15K SNPs were used for genomic prediction. The population structure associated with the 1B.1R translocation was described in earlier publications (Lopes *et al.* 2015; Sukumaran *et al.* 2015a).

### Statistical models

The Best Linear Unbiased Estimators (BLUEs) were computed for mixed model analysis for each of the traits in each environment. The model used to calculate BLUEs for each environment is

$$y_{jkm}=\mu +L_{j}+r_{k}+b_{m\left(k\right)}+e_{jkm}$$

where $y_{jkm}$ is the phenotypic response value for the specific trait measured on the *j*th line of the *m*th incomplete block within the *k*th replicate, $L_{j}$ is a fixed effect of the *j*th wheat line, $r_{k}$ is the random effect of the *k*th replicate assumed independent and identically multivariate normally distributed (iid) *N*(0, $I\sigma_{r}^{2}$) (where ***I*** is the identity matrix, and $\sigma_{r}^{2}$ is the variance of replicate), $b_{m\left(k\right)}$ denotes the random effect of the *m*th incomplete block within the *k*th replicate assumed independent and identically distributed (iid) with *N*(0, $I\sigma_{b}^{2}$), where $\sigma_{b}^{2}$ is the variance of the incomplete block, $e_{jkm}$ is the random error associated to the trait measured on the *j*th line of the *m*th incomplete block within the *k*th replicate, and assumed iid with *N*(0, $I\sigma_{e}^{2}$), where $\sigma_{e}^{2}$ denotes the error variance.

Broad-sense heritability (*H*^2^) for each environment was computed on an entry mean basis as ${\text{H}}^{2}=\left[\sigma_{g}^{2}/\sigma_{g}^{2}+\left(\frac{\sigma_{e}^{2}}{r}\right)\right],$ where $\sigma_{g}^{2}$ is the wheat line variance, *r* is the number of replicates, and $\sigma_{e}^{2}$ is the error variance. Heritability estimates across environments were also estimated using the following formula,

$${\text{H}}^{2}=\frac{\sigma_{g}^{2}}{\sigma_{g}^{2}+\frac{\sigma_{ge}^{2}}{s}+\frac{\sigma_{e}^{2}}{rs}}$$

where *s* is the number of environments, and $\sigma_{ge}^{2}$ is the variance of the wheat line × environment obtained from the combined analyses across environments.

For GS, we used the reaction norm model that is an extension of the random effect Genomic Best Linear Unbiased Predictor (GBLUP) model, where the main effect of lines, the main effect of environments, the main effect of markers, the main effect of pedigree, and their interactions with environments, are modeled using random covariance structures that are functions of marker or pedigree genotypes and environmental covariates (Jarquín *et al.* 2014). Brief descriptions of the baseline model, as well as the reaction norm models with G×E, are given below.

### Baseline model

The response of the phenotypes ($y_{ij}$) defined by random baseline model is

$$y_{ij}=\mu +E_{i}+L_{j}+EL_{ij}+e_{ij}$$

where μ is the overall mean, $E_{i}$ is the random effect of the *i*th environment, $L_{j}$ is the random effect of the *j*th line, $EL_{ij}$ is the interaction between the *i*th environment and the *j*th line, and $e_{ij}$ is the random error term. All random effects follow a iid multivariate normal distribution such that $E_{i}∼N\left(0,I\sigma_{E}^{2}\right),$ $L_{j}∼N\left(0,I\sigma_{L}^{2}\right),$ $EL_{ij}∼N\left(0,I\sigma_{EL}^{2}\right),$ and $e_{ij}∼N\left(0,I\sigma_{e}^{2}\right)$ where $\sigma_{E}^{2},$ $\sigma_{L}^{2},$ and $\sigma_{EL}^{2}$ are the environment, line, and line × environment variances, respectively.

In the model above, the random effect of the line ($L_{j}$) can be replaced by $g_{j},$ which is an approximation of the genetic value of the *j*th line from the genomic relationship matrix. Also, the effects of the line ($L_{j}$) can be replaced by $a_{j},$ which is the additive effect obtained from the pedigree information. In the models described below, we used either $g_{j}$ or $a_{j},$ both $g_{j}$ and $a_{j}$, as well as their interactions with environment $E_{i}\left(gE_{ij},\text{or}\;aE_{ij}\right).$ Full descriptions of the different reaction norm models can be found in Jarquín *et al.* (2014) and Zhang *et al.* (2014), among others. Below, we give a brief description of the different reaction norm models that were fitted using pedigree and genomic information.

### Reaction norm models

We fitted seven different models (M1–M7) with different components including *E* = environments, *L* = line, *A* = pedigree, *G* = genomic, *AE* = pedigree × environment interaction, *GE* = genomic × environment interaction, and *e* = residual error.

### M1: Environment and line main effects (Y = E + L + e)

The response of the phenotypes ($y_{ij}$) from the baseline model, but excluding the interaction term, $EL_{ij},$ is described as

$$y_{ij}=\mu +E_{i}+L_{j}+e_{ij}$$ (1)

### M2: Environment, line, and pedigree main effects (Y = E + L + A + e)

By adding the random effect that incorporates pedigree information by means of the numerical relationship matrix (***A***) to M1, we get model M2, defined as

$$y_{ij}=\mu +E_{i}+L_{j}+a_{j}+e_{ij}$$ (2)

where $a_{j}$ is a random additive effect of the line, which, in this case accounts for pedigree-relationships, where $a={\left(a,\ldots ,a_{J}\right)}^{\prime }$ contains the pedigree values of all the lines, and is assumed to follow a multivariate normal density with zero mean and covariance matrix $Cov\left(a\right)=A\sigma_{a}^{2},$ where ***A*** is the numerical relationship matrix, and $\sigma_{a}^{2}$ is the additive genetic variance. The random effects $a={\left(a,\ldots ,a_{J}\right)}^{\prime }$ are correlated such that model M2 allows borrowing of information across lines based on the numerical relationship matrix (***A***) computed from the pedigree information.

### M3: Environment, line, and genomic main effects (Y = E + L + G + e)

Model M3 is fitted by adding the genomic random effect of the line $g_{j}$ to M1, which is an approximation of the genetic value of the *j*th line, and is defined by the regression on marker covariates $g_{j}=\sum_{l=1}^{p}x_{jl}c_{l},$ where $x_{jl}$ is the genotype of the *j*th line at the *l*th marker, and $c_{l}$ is the effect of the *l*th marker assuming iid $c_{l}∼N\left(0,\sigma_{c}^{2}\right)$ (*l*=1,…,*p*), and $\sigma_{c}^{2}$ is the variance of the marker effects. The vector $g={\left(g_{1},\ldots ,g_{J}\right)}^{\prime }$ contains the genomic values of all the lines, and is assumed to follow a multivariate normal density with zero mean and covariance matrix $Cov\left(g\right)=G\sigma_{g}^{2},$ where ***G*** is the genomic relationship matrix computed as suggested by VanRaden (2008) (*i.e.*, $G\propto (XX^{\prime }/2\sum_{l=1}^{p}p_{l}(1-p_{l})),$ with X as the centered and standardized matrix of molecular markers and $p_{l}$ the frequency of the *l*th marker); and $\sigma_{g}^{2}\propto \sigma_{c}^{2}$ is the genomic variance. Thus, model M3 is

$$y_{ij}=\mu +E_{i}+L_{j}+g_{j}+e_{ij}$$ (3)

with $g∼N\left(0,G\sigma_{g}^{2}\right).$ The random effects $g={\left(g_{1},\ldots ,g_{J}\right)}^{\prime }$ are correlated such that model M3 allows borrowing information across lines.

### M4: Environment, line, pedigree, and pedigree × environment interaction effects (Y = E + L + A + AE + e)

By adding the interaction between the additive relationship matrix and environments ($Ea_{ij}$) to model M2, model M4 becomes

$$y_{ij}=\mu +E_{i}+L_{j}+a_{j}+Ea_{ij}+e_{ij},$$ (4)

where the term $Ea_{ij}$ is the interaction between the additive value of the *i*th genotype in the *j*th environment and $\mathrm{Ea}∼N\left[0,\left(Z_{a}GZ_{a}^{\prime }\right){}^{\circ}\left(Z_{E}Z_{E}^{\prime }\right)\sigma_{\mathrm{Ea}}^{2}\right].$ Matrices $Z_{a}$ and $Z_{E}$ are the incidence matrices for the effects of the additive genetic values of genotypes and environments, respectively, $\sigma_{Ea}^{2}$ is the variance component of the interaction term $Ea_{ij}$, and “∘” stands for Hadamart product between two matrices.

### M5: Environments, lines, genomic, genomic × environment interaction effect (genomic × environment) (Y = E + L + G + GE + e)

By adding the interaction between markers and environments ($Eg_{ij}$) to model M3, model M5 becomes

$$y_{ij}=\mu +E_{i}+L_{j}+g_{j}+Eg_{ij}+e_{ij},$$ (5)

where the term $Eg_{ij}$ is the interaction between the genetic value of the *i*th genotype in the *j*th environment; then $\mathrm{Eg}∼N[0,(Z_{g}GZ^{\prime }{}_{g}){}^{\circ}(Z_{E}Z^{\prime }{}_{E})[\sigma_{\mathrm{Eg}}^{2}].$ Matrices $Z_{g}$ and $Z_{E}$ are the incidence matrices for the effects of the genetic values of the genotypes and the environments, respectively, $\sigma_{Eg}^{2}$ is the variance component of the interaction term $Eg_{ij}.$

### M6: Environment, line, pedigree, and genomic main effects (Y = E + L + A + G + e)

We added both the pedigree and genomic effects of the lines ($g_{j},$ and $a_{j}$) to model M1, so that it contains the genomic random vector $g=(g_{1},...,g_{J}{)}^{\prime },$ and the pedigree random vector $a=(a,...,a{)}^{\prime }.$ Therefore, model M6 is

$$y_{ij}=\mu +E_{i}+L_{j}+g_{j}+a_{j}+e_{ij},$$ (6)

### M7: Environment, line, pedigree, genomic, pedigree × environment interaction and genomic × environment interaction effects (M7 = E + L + A + G + AE + GE + e)

By adding both the interaction between pedigree and environment ($Ea_{ij}$), and the interaction between markers (genomic) and environments ($Eg_{ij}$) to model M6, model M7 becomes

$$y_{ij}=\mu +E_{i}+L_{j}+g_{j}+a_{j}+Eg_{ij}+Ea_{ij}+e_{ij},$$ (7)

All the terms in this model have already been defined above.

### Prediction assessment by cross-validation

Two distinct cross-validation (CV) schemes were used. The first, CV1, evaluates the prediction ability of models when a set of lines has not been evaluated in any of the environments (Burgueño *et al.* 2012). Predictions derived using CV1 are based entirely on phenotypic records of other lines. The second scheme, CV2, evaluates the prediction ability of models when some lines have been evaluated in some environments, but not in others. In CV2 prediction, information from related lines and the correlated environments is used, and prediction assessment benefits from borrowing information between lines within an environment, between lines across environments, and among correlated environments (Burgueño *et al.* 2012). Prediction ability is the Pearson correlation coefficient between the observed and predicted values for each genotype.

In both CV1 and CV2, a fivefold cross-validation scheme was used to generate the training (TRN) and testing (TST) sets, and to assess the prediction ability of each testing set. The data were divided randomly into five subsets, with 80% of the lines assigned to the training set and 20% assigned to the testing set. Four subsets were combined to form the training set, and the remaining subset was used as the validation set. Permutation of five subsets led to five possible training and validation data sets. This procedure was repeated 20 times, and a total of 100 runs was performed on each population for each trait-environment combination. The same partitions were analyzed with all models. The average value of the correlations between the phenotype and the genomic estimated breeding values from 100 runs was calculated in each population for each trait-environment combination, and was defined as the prediction ability.

### Software

The genomic prediction analyses were computed using R, and the models were fitted using the BGLR package (de los Campos *et al.* 2013; de los Campos and Pérez-Rodríguez 2013). The ANOVA was performed in the SAS 9.2 (SAS Institute Inc 2010) program, and the boxplots created in R. Tukey’s test for significant differences between the models’ predictions (correlations) were generated in the SAS 9.2 (SAS Institute Inc 2010) program.

### Data availability

All the phenotypic data for each environment and trait, as well as the genomic data, can be downloaded from the link http://hdl.handle.net/11529/10714. 

## Results

### Variation in the studied traits

**Figure 2:**
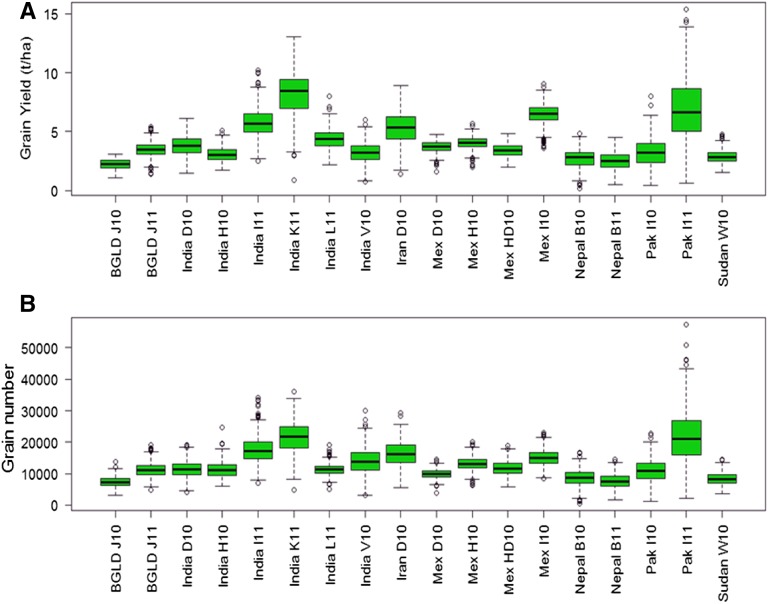
Boxplot of the data collected from 18 environments around the world for traits (A) GY and (B) GN. Environments (site-year combinations) are defined in Table 1.

**Figure 3:**
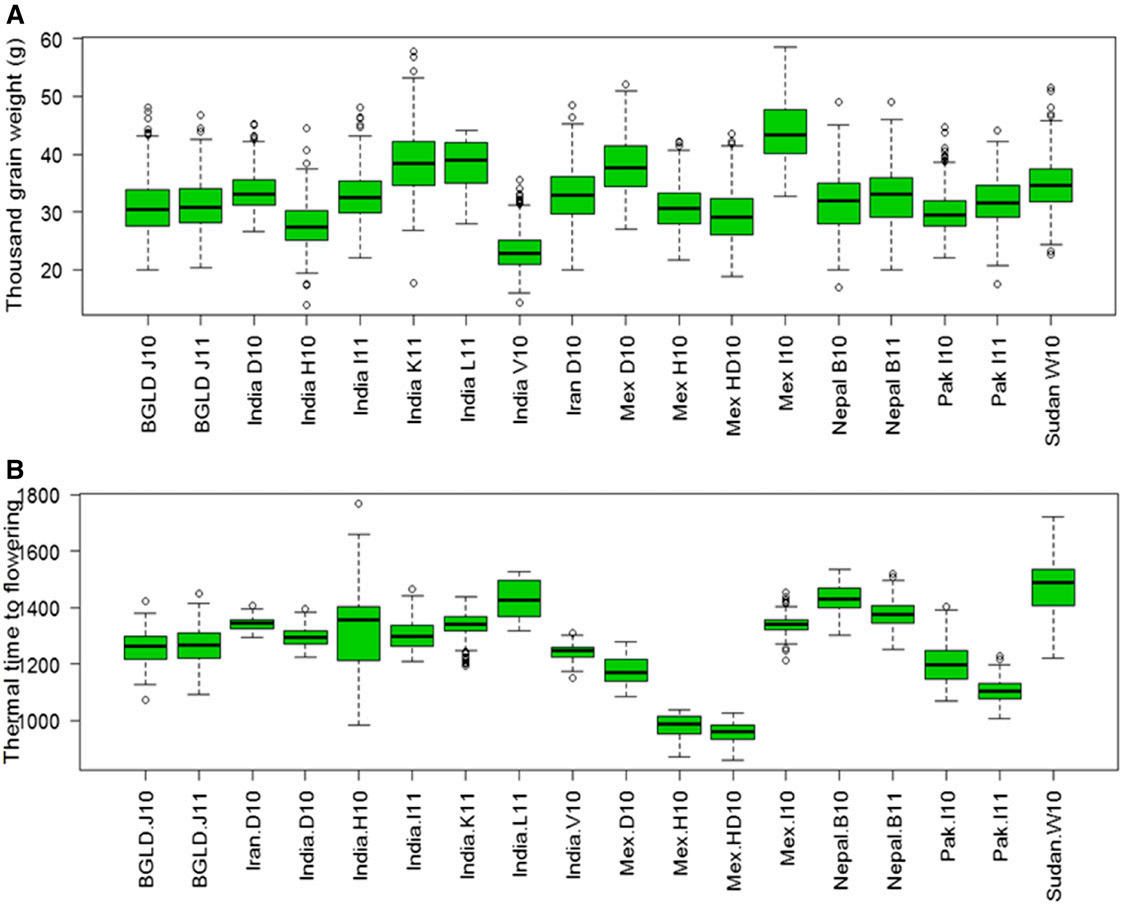
Boxplot of the data collected from 18 environments around the world for traits (A) GW and (B) TTF. Environments (site-year combinations) are defined in Table 1.

The WAMI panel was grown in seven countries, comprising a total of 18 environments (site-year combinations). India had the largest number of environments (6) followed by Mexico (4), and Bangladesh, Nepal, Pakistan, Iran, and Sudan with one site each. The ANOVA showed significant differences between the wheat lines and environments (Table A1, Appendix A). Environment Mex 110 was the highest yielding environment (7.02 ton/ha), followed by Pak I11 (6.9 ton/ha). The lowest yield was obtained in BGLD J10 (2.2 ton/ha) with a GW value of 30.9. The highest GW value was recorded in Mex I10 (43.4), followed by India L11 (38.3), and the lowest was India H10 (27.6). TTF ranged from 976°D in Mex H10 to 1474°D in Sudan W. The highest GN was recorded in Pak I11 (21920), followed by Iran D10 (Table 1). Heritability estimates of individual and combined environments for each trait were also calculated. Trait GW (0.74) had the highest *H*^2^ values, followed by GN (0.51), TTF (0.48), and GY (0.41) (Table A1, Appendix A). A box plot of the data at the individual locations indicated TTF had the highest variation and the lowest G × E was observed for GW (Figure 2 and Figure 3).

### Prediction ability of different models for international sites

**Figure 4:**
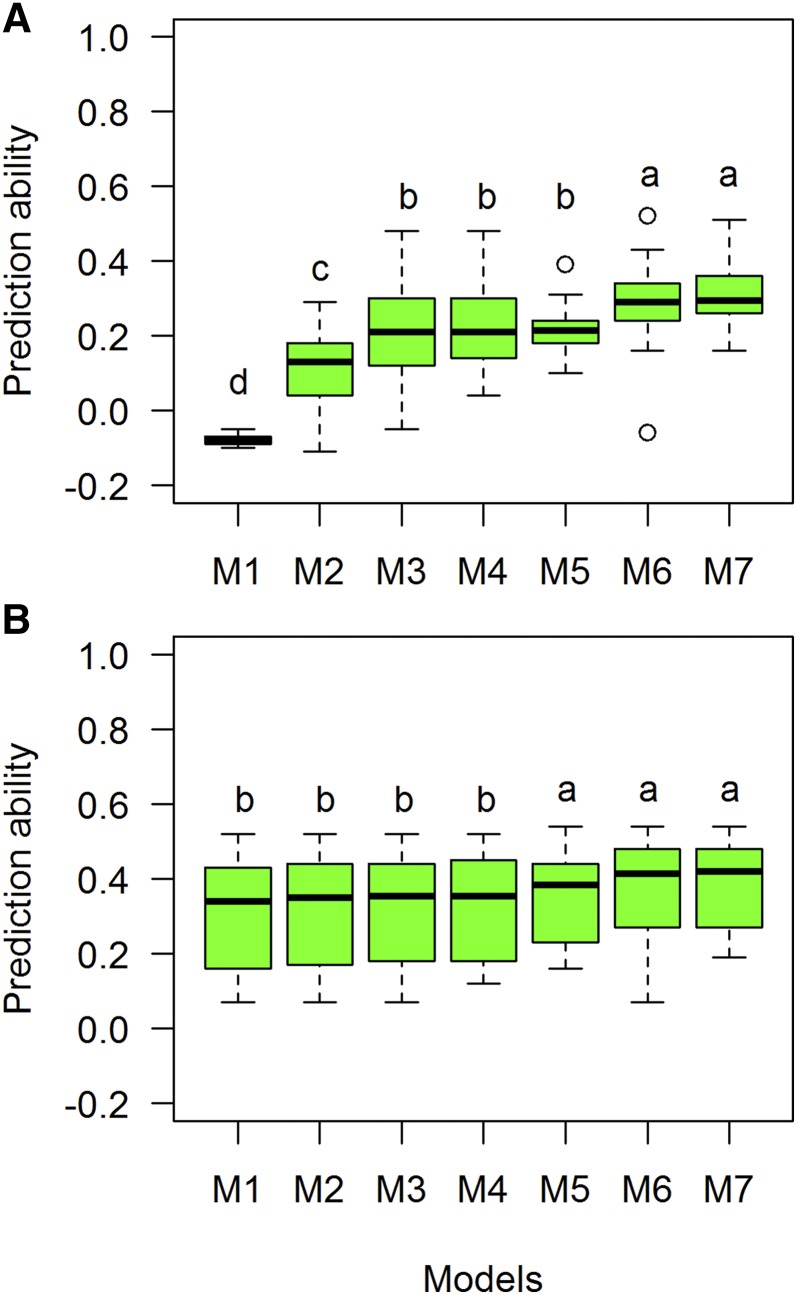
Comparison of boxplot distributions of prediction ability (correlations) for each model (M1–M7) for trait GY using two prediction CV scenarios: (A) CV1 and (B) CV2 for GY. Different letters denote significant differences among groups (*post hoc* nonparametric Tukey’s test, *P* < 0.05). Models: M1 Y = E+L+e; M2 Y= E+L+A+e; M3 Y= E+L+G+e; M4 Y= E+L+A+AE+e; M5 Y= E+L+G+GE+e; M6 Y= E+L+G+A+e; M7 Y= E+L+G+A+GE+AE+e.

We used seven models (M1–M7) to predict the lines that were not observed under CV1 and CV2 scenarios. For GY, prediction ability values for individual sites ranged from −0.05 (M1) to 0.52 (M6) (Table 2). The highest value obtained for CV1 was in Mex D10 (0.52). The models’ average prediction abilities were −0.08, 0.11, 0.21, 0.22, 0.22, 0.29, and 0.31 for M1–M7, respectively. Models 6 and 7 had the highest significant prediction ability, followed by models 3, 4, and 5 (Figure 4). For CV2, the values ranged from 0.10 (Sudan W10) to 0.54 (India D10, India V10). On average, the CV2 correlation values for each model were 0.31, 0.31, 0.32, 0.32, 0.35, 0.37, and 0.38 for M1–M7, respectively (Table 2). Tukey’s test identified that M5–M7 had significantly higher prediction accuracies than the other models for CV2 (Figure 4).

**Table 2:** Correlations (mean ± SD) between the observed and predicted values for GY under CV1 and CV2 CV schemes for seven prediction models (M1–M7)

	CV1							CV2							*H*^2^
Env\Model	M1*^a^*	M2	M3	M4	M5	M6	M7	M1	M2	M3	M4	M5	M6	M7	
BGLD J10*^b^*	−0.06 ± 0.05	0.04 ± 0.02	0.09 ± 0.02	0.09 ± 0.02	0.22 ± 0.03	0.16 ± 0.02	0.21 ± 0.03	0.21 ± 0.01	0.20 ± 0.01	0.20 ± 0.01	0.20 ± 0.01	0.26 ± 0.02	0.24 ± 0.02	0.26 ± 0.02	0.20
BGLD J11	−0.08 ± 0.07	0.00 ± 0.03	0.05 ± 0.02	0.06 ± 0.02	0.22 ± 0.03	0.29 ± 0.02	0.30 ± 0.03	0.30 ± 0.01	0.30 ± 0.01	0.29 ± 0.01	0.29 ± 0.01	0.38 ± 0.01	0.42 ± 0.01	0.43 ± 0.01	0.67
India D10	−0.09 ± 0.08	0.13 ± 0.05	0.21 ± 0.03	0.22 ± 0.03	0.24 ± 0.03	0.30 ± 0.03	0.30 ± 0.03	0.52 ± 0.01	0.52 ± 0.01	0.52 ± 0.01	0.52 ± 0.01	0.54 ± 0.01	0.54 ± 0.01	0.54 ± 0.01	0.55
India H10	−0.06 ± 0.06	−0.11 ± 0.04	−0.05 ± 0.02	0.14 ± 0.03	0.20 ± 0.03	−0.06 ± 0.03	0.21 ± 0.03	0.07 ± 0.02	0.07 ± 0.01	0.07 ± 0.01	0.16 ± 0.02	0.19 ± 0.02	0.07 ± 0.01	0.20 ± 0.02	0.13
India I11	−0.10 ± 0.05	0.18 ± 0.06	0.32 ± 0.03	0.32 ± 0.03	0.23 ± 0.02	0.31 ± 0.02	0.30 ± 0.02	0.39 ± 0.02	0.40 ± 0.02	0.41 ± 0.02	0.41 ± 0.02	0.40 ± 0.02	0.42 ± 0.02	0.41 ± 0.02	0.44
India K10	−0.08 ± 0.05	0.05 ± 0.03	0.16 ± 0.04	0.15 ± 0.03	0.18 ± 0.03	0.28 ± 0.02	0.26 ± 0.02	0.15 ± 0.02	0.16 ± 0.02	0.17 ± 0.02	0.17 ± 0.02	0.23 ± 0.02	0.31 ± 0.02	0.29 ± 0.02	0.28
India L11	−0.10 ± 0.05	0.04 ± 0.04	0.20 ± 0.03	0.19 ± 0.03	0.19 ± 0.04	0.26 ± 0.03	0.29 ± 0.03	0.36 ± 0.02	0.36 ± 0.02	0.37 ± 0.02	0.36 ± 0.02	0.41 ± 0.01	0.41 ± 0.02	0.43 ± 0.02	0.79
India V10	−0.09 ± 0.05	0.14 ± 0.05	0.21 ± 0.03	0.22 ± 0.03	0.24 ± 0.03	0.29 ± 0.04	0.29 ± 0.03	0.52 ± 0.01	0.52 ± 0.01	0.52 ± 0.01	0.52 ± 0.01	0.54 ± 0.01	0.53 ± 0.01	0.53 ± 0.02	0.55
Iran D10	−0.10 ± 0.06	0.06 ± 0.08	0.03 ± 0.01	0.04 ± 0.04	0.18 ± 0.04	0.24 ± 0.01	0.26 ± 0.03	0.11 ± 0.02	0.12 ± 0.02	0.11 ± 0.02	0.12 ± 0.02	0.19 ± 0.02	0.26 ± 0.02	0.27 ± 0.02	0.97
Mex D10	−0.05 ± 0.07	0.29 ± 0.03	0.48 ± 0.02	0.48 ± 0.02	0.39 ± 0.02	0.52 ± 0.02	0.51 ± 0.02	0.43 ± 0.01	0.44 ± 0.01	0.46 ± 0.01	0.46 ± 0.01	0.50 ± 0.01	0.54 ± 0.01	0.53 ± 0.01	0.68
Mex H10	−0.06 ± 0.06	0.18 ± 0.05	0.36 ± 0.02	0.35 ± 0.02	0.21 ± 0.05	0.41 ± 0.03	0.41 ± 0.03	0.44 ± 0.01	0.44 ± 0.01	0.45 ± 0.01	0.45 ± 0.01	0.44 ± 0.01	0.48 ± 0.01	0.48 ± 0.02	0.76
Mex HD10	−0.07 ± 0.08	0.16 ± 0.04	0.30 ± 0.02	0.30 ± 0.02	0.21 ± 0.04	0.34 ± 0.03	0.35 ± 0.03	0.39 ± 0.02	0.40 ± 0.02	0.41 ± 0.02	0.41 ± 0.02	0.41 ± 0.02	0.43 ± 0.02	0.43 ± 0.02	0.60
Mex I10	−0.08 ± 0.05	0.24 ± 0.03	0.34 ± 0.03	0.35 ± 0.03	0.29 ± 0.02	0.43 ± 0.03	0.43 ± 0.03	0.35 ± 0.01	0.36 ± 0.01	0.37 ± 0.01	0.37 ± 0.01	0.39 ± 0.01	0.43 ± 0.01	0.43 ± 0.01	0.74
Nepal B10	−0.08 ± 0.04	0.20 ± 0.03	0.28 ± 0.02	0.29 ± 0.02	0.17 ± 0.03	0.26 ± 0.02	0.28 ± 0.02	0.33 ± 0.02	0.34 ± 0.02	0.34 ± 0.02	0.35 ± 0.02	0.33 ± 0.02	0.35 ± 0.02	0.36 ± 0.02	0.41
Nepal B11	−0.08 ± 0.06	0.15 ± 0.03	0.24 ± 0.02	0.25 ± 0.02	0.31 ± 0.02	0.33 ± 0.03	0.36 ± 0.03	0.44 ± 0.01	0.44 ± 0.01	0.44 ± 0.01	0.45 ± 0.01	0.49 ± 0.01	0.50 ± 0.01	0.51 ± 0.01	0.55
Pak I10	−0.09 ± 0.06	0.08 ± 0.04	0.21 ± 0.03	0.20 ± 0.03	0.10 ± 0.03	0.23 ± 0.02	0.20 ± 0.03	0.26 ± 0.02	0.27 ± 0.02	0.27 ± 0.02	0.27 ± 0.01	0.26 ± 0.02	0.30 ± 0.02	0.28 ± 0.02	0.33
Pak I11	−0.10 ± 0.06	0.02 ± 0.08	0.12 ± 0.06	0.13 ± 0.06	0.10 ± 0.04	0.17 ± 0.04	0.16 ± 0.03	0.16 ± 0.03	0.17 ± 0.03	0.18 ± 0.03	0.18 ± 0.03	0.16 ± 0.02	0.20 ± 0.03	0.19 ± 0.03	0.26
Sudan W10	−0.07 ± 0.06	0.13 ± 0.03	0.15 ± 0.02	0.16 ± 0.02	0.31 ± 0.03	0.38 ± 0.02	0.38 ± 0.02	0.10 ± 0.02	0.11 ± 0.02	0.11 ± 0.02	0.12 ± 0.02	0.21 ± 0.02	0.27 ± 0.02	0.27 ± 0.02	0.30
Average	−0.08	0.11	0.21	0.22	0.22	0.29	0.31	0.31	0.31	0.32	0.32	0.35	0.37	0.38	

Broad-sense heritability *H*^2^ of GY in each environment.

a Models: M1 Y = E+L+e; M2 Y= E+L+A+e; M3 Y= E+L+G+e; M4 Y= E+L+A+AE+e; M5 Y= E+L+G+GE+e; M6 Y= E+L+G+A+e; M7 Y= E+L+G+A+GE+AE+e.

b Names of the environments are given in Table 1.

For CV1, 28 environment-model combinations had prediction ability values above 0.30 for GY. Among the sites, when using M7, Mexican environments (Mex I10, Mex D10, and Mex H10) had high prediction ability values (>0.41) for CV1 using M6 and M7. CV2 values were above 0.40 for 52 site-model combinations. For India V10 and India D10, prediction ability was above 0.5 for all models. In the CV1 scenario, 14 sites had the highest values when M6 and M7 were used (Table 2).

**Figure 5:**
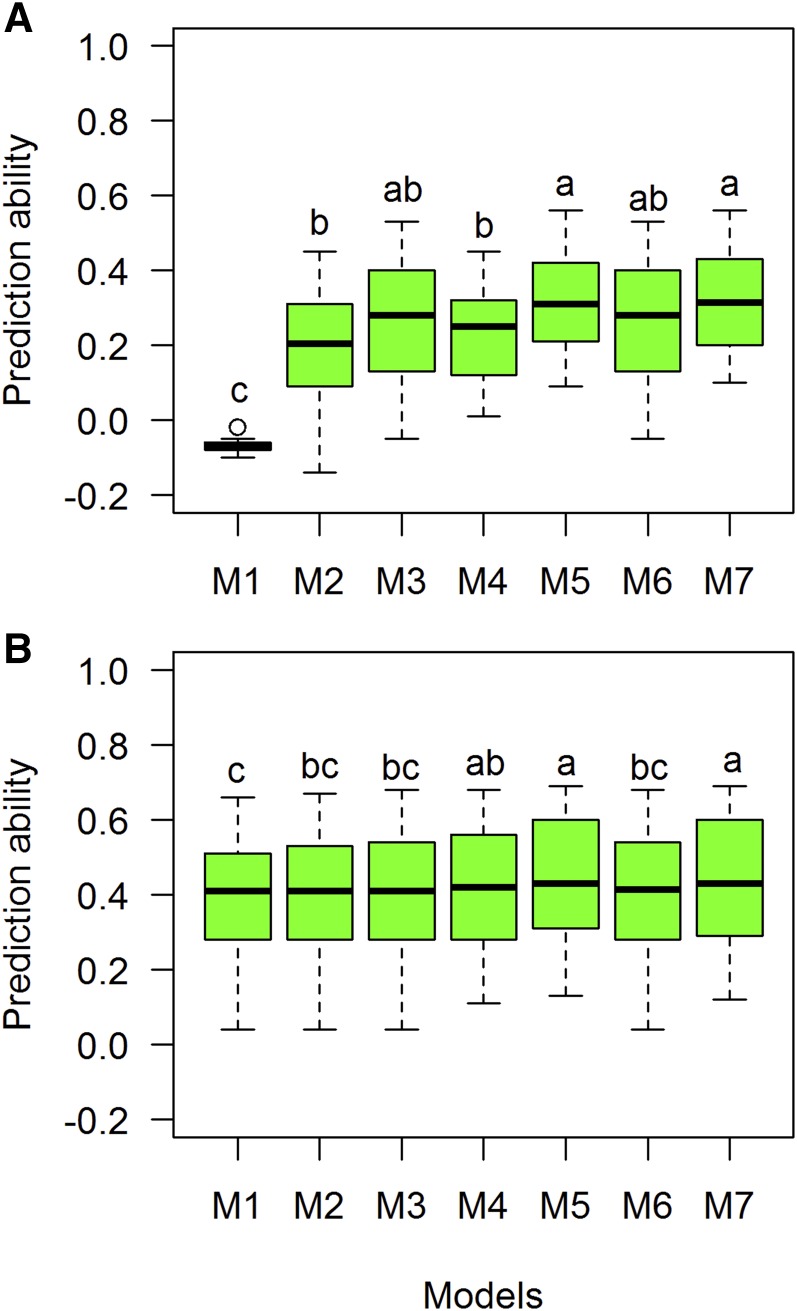
Comparison of boxplot distributions of prediction ability (correlations) for each model (M1–M7) for trait GN using two prediction CV scenarios: (A) CV1 and (B) CV2 for GY. Different letters denote significant differences among groups (*post hoc* nonparametric Tukey’s test, *P* < 0.05). Models: M1 Y = E+L+e; M2 Y= E+L+A+e; M3 Y= E+L+G+e; M4 Y= E+L+A+AE+e; M5 Y= E+L+G+GE+e; M6 Y= E+L+G+A+e; M7 Y= E+L+G+A+GE+AE+e.

Trait GN mostly followed a similar pattern as that shown for GY but the CV1 values ranged from −0.05 (BGLD J10) to 0.56 (Mex I10). Forty-three environment-model combinations had CV1 values >0.30. Models 6 and 7 had five sites with CV1 values >0.4. Two sites (Sudan W10 and Mex I10) had CV1 values > 0.4 for six models. Models 6 and 7 had CV1 values > 0.50 for four environments (Mex H10, Mex HD10, Sudan W10, and Mex I10). On average, the highest CV1 values were recorded for M5 (0.32) and M7 (0.32) (Table 3). Tukey’s test also grouped M5 and M7 with the highest prediction ability models for CV1 and CV2 scenarios (Figure 5).

**Table 3:** Correlations (mean ± SD) between the observed and predicted values for GN under CV1 and CV2 schemes for seven prediction models (M1–M7)

	CV1							CV2							*H*^2^
Env\Model	M1*^a^*	M2	M3	M4	M5	M6	M7	M1	M2	M3	M4	M5	M6	M7	
BGLD J10*^b^*	−0.05 ± 0.05	0.07 ± 0.01	0.13 ± 0.01	0.01 ± 0.03	0.09 ± 0.04	0.13 ± 0.02	0.10 ± 0.04	0.26 ± 0.01	0.26 ± 0.01	0.26 ± 0.01	0.26 ± 0.02	0.25 ± 0.01	0.26 ± 0.01	0.25 ± 0.01	0.26
BGLD J11	−0.07 ± 0.06	0.15 ± 0.01	0.22 ± 0.01	0.12 ± 0.02	0.21 ± 0.02	0.22 ± 0.01	0.20 ± 0.03	0.43 ± 0.01	0.42 ± 0.01	0.42 ± 0.01	0.42 ± 0.01	0.43 ± 0.01	0.42 ± 0.01	0.42 ± 0.01	0.68
India D10	−0.08 ± 0.06	0.23 ± 0.03	0.31 ± 0.01	0.25 ± 0.03	0.32 ± 0.03	0.31 ± 0.01	0.33 ± 0.03	0.42 ± 0.01	0.43 ± 0.01	0.43 ± 0.01	0.44 ± 0.01	0.43 ± 0.01	0.43 ± 0.01	0.44 ± 0.01	0.50
India H10	−0.06 ± 0.07	0.22 ± 0.02	0.30 ± 0.02	0.30 ± 0.02	0.37 ± 0.02	0.29 ± 0.02	0.38 ± 0.03	0.35 ± 0.01	0.36 ± 0.01	0.37 ± 0.01	0.42 ± 0.01	0.43 ± 0.01	0.37 ± 0.01	0.44 ± 0.01	0.40
India I11	−0.08 ± 0.07	−0.08 ± 0.02	−0.05 ± 0.02	0.17 ± 0.04	0.21 ± 0.03	−0.05 ± 0.02	0.20 ± 0.03	0.04 ± 0.02	0.04 ± 0.02	0.04 ± 0.02	0.17 ± 0.03	0.21 ± 0.02	0.04 ± 0.02	0.22 ± 0.03	0.40
India K10	−0.08 ± 0.06	0.15 ± 0.03	0.23 ± 0.02	0.14 ± 0.03	0.21 ± 0.03	0.22 ± 0.02	0.20 ± 0.03	0.39 ± 0.02	0.39 ± 0.02	0.39 ± 0.02	0.37 ± 0.02	0.37 ± 0.02	0.39 ± 0.02	0.36 ± 0.03	0.28
India L11	−0.09 ± 0.05	−0.14 ± 0.01	−0.02 ± 0.01	0.10 ± 0.04	0.21 ± 0.03	−0.04 ± 0.01	0.23 ± 0.03	0.06 ± 0.01	0.04 ± 0.01	0.05 ± 0.01	0.14 ± 0.02	0.17 ± 0.02	0.05 ± 0.01	0.19 ± 0.02	0.72
India V10	−0.06 ± 0.05	0.20 ± 0.02	0.27 ± 0.02	0.24 ± 0.02	0.32 ± 0.02	0.27 ± 0.02	0.32 ± 0.02	0.37 ± 0.01	0.38 ± 0.01	0.38 ± 0.01	0.39 ± 0.01	0.41 ± 0.02	0.38 ± 0.01	0.41 ± 0.01	0.48
Iran D10	−0.10 ± 0.06	0.21 ± 0.04	0.29 ± 0.02	0.25 ± 0.03	0.30 ± 0.03	0.29 ± 0.02	0.31 ± 0.03	0.40 ± 0.01	0.40 ± 0.01	0.41 ± 0.01	0.42 ± 0.01	0.41 ± 0.02	0.41 ± 0.01	0.41 ± 0.02	0
Mex D10	−0.02 ± 0.06	0.30 ± 0.02	0.40 ± 0.01	0.32 ± 0.02	0.42 ± 0.01	0.40 ± 0.01	0.43 ± 0.01	0.60 ± 0.01	0.61 ± 0.01	0.61 ± 0.01	0.61 ± 0.01	0.62 ± 0.01	0.61 ± 0.01	0.63 ± 0.01	0.74
Mex H10	−0.06 ± 0.04	0.36 ± 0.02	0.51 ± 0.01	0.37 ± 0.01	0.52 ± 0.01	0.50 ± 0.01	0.51 ± 0.01	0.66 ± 0.01	0.67 ± 0.01	0.68 ± 0.01	0.67 ± 0.01	0.69 ± 0.01	0.68 ± 0.01	0.69 ± 0.01	0.84
Mex HD10	−0.07 ± 0.06	0.38 ± 0.02	0.51 ± 0.01	0.42 ± 0.01	0.53 ± 0.01	0.50 ± 0.01	0.53 ± 0.01	0.65 ± 0.01	0.66 ± 0.01	0.67 ± 0.01	0.68 ± 0.01	0.69 ± 0.01	0.67 ± 0.01	0.69 ± 0.01	0.74
Mex I10	−0.05 ± 0.05	0.41 ± 0.02	0.53 ± 0.01	0.43 ± 0.02	0.56 ± 0.01	0.53 ± 0.01	0.56 ± 0.01	0.64 ± 0.01	0.65 ± 0.01	0.66 ± 0.01	0.66 ± 0.01	0.69 ± 0.01	0.66 ± 0.01	0.68 ± 0.01	0.83
Nepal B10	−0.07 ± 0.05	0.31 ± 0.02	0.32 ± 0.01	0.29 ± 0.01	0.32 ± 0.02	0.34 ± 0.01	0.34 ± 0.01	0.46 ± 0.01	0.47 ± 0.01	0.47 ± 0.01	0.46 ± 0.01	0.47 ± 0.02	0.47 ± 0.01	0.47 ± 0.01	0.48
Nepal B11	−0.06 ± 0.06	0.14 ± 0.01	0.08 ± 0.01	0.27 ± 0.02	0.20 ± 0.03	0.11 ± 0.02	0.24 ± 0.03	0.44 ± 0.01	0.43 ± 0.01	0.41 ± 0.01	0.49 ± 0.01	0.50 ± 0.01	0.42 ± 0.01	0.51 ± 0.01	0.50
Pak I10	−0.08 ± 0.07	0.09 ± 0.02	0.15 ± 0.02	0.10 ± 0.03	0.20 ± 0.02	0.14 ± 0.02	0.18 ± 0.02	0.28 ± 0.01	0.28 ± 0.01	0.28 ± 0.01	0.28 ± 0.02	0.31 ± 0.02	0.28 ± 0.01	0.29 ± 0.02	0.36
Pak I11	−0.10 ± 0.06	0.02 ± 0.05	0.06 ± 0.04	0.08 ± 0.04	0.12 ± 0.04	0.06 ± 0.04	0.11 ± 0.04	0.08 ± 0.02	0.08 ± 0.02	0.09 ± 0.02	0.11 ± 0.03	0.13 ± 0.02	0.08 ± 0.02	0.12 ± 0.02	0.25
Sudan W10	−0.07 ± 0.07	0.45 ± 0.02	0.53 ± 0.01	0.45 ± 0.02	0.56 ± 0.01	0.53 ± 0.01	0.55 ± 0.01	0.51 ± 0.01	0.53 ± 0.01	0.54 ± 0.01	0.56 ± 0.01	0.60 ± 0.01	0.54 ± 0.01	0.60 ± 0.01	0.59
Average	−0.07	0.19	0.27	0.24	0.32	0.26	0.32	0.39	0.39	0.40	0.42	0.43	0.40	0.43	

Broad-sense heritability *H*^2^ of GN in each environment.

a Models: M1 Y = E+L+e; M2 Y= E+L+A+e; M3 Y= E+L+G+e; M4 Y= E+L+A+AE+e; M5 Y= E+L+G+GE+e; M6 Y= E+L+G+A+e; M7 Y= E+L+G+A+GE+AE+e.

b Names of the environments are given in Table 1.

For CV2, 36 environment-model combinations had GN values >0.5. The values ranged from 0.08 (Iran D10) to 0.69 (Mex HD10). Five environments (Mex D10, Mex H10, Mex HD10, Sudan W10, and Mex I10) had values >0.5 for all models. On average, the increase in CV2 values from M1 to M7 was 0.04, with M5 and M7 recording the highest average increases (0.43) (Table 3).

**Figure 6:**
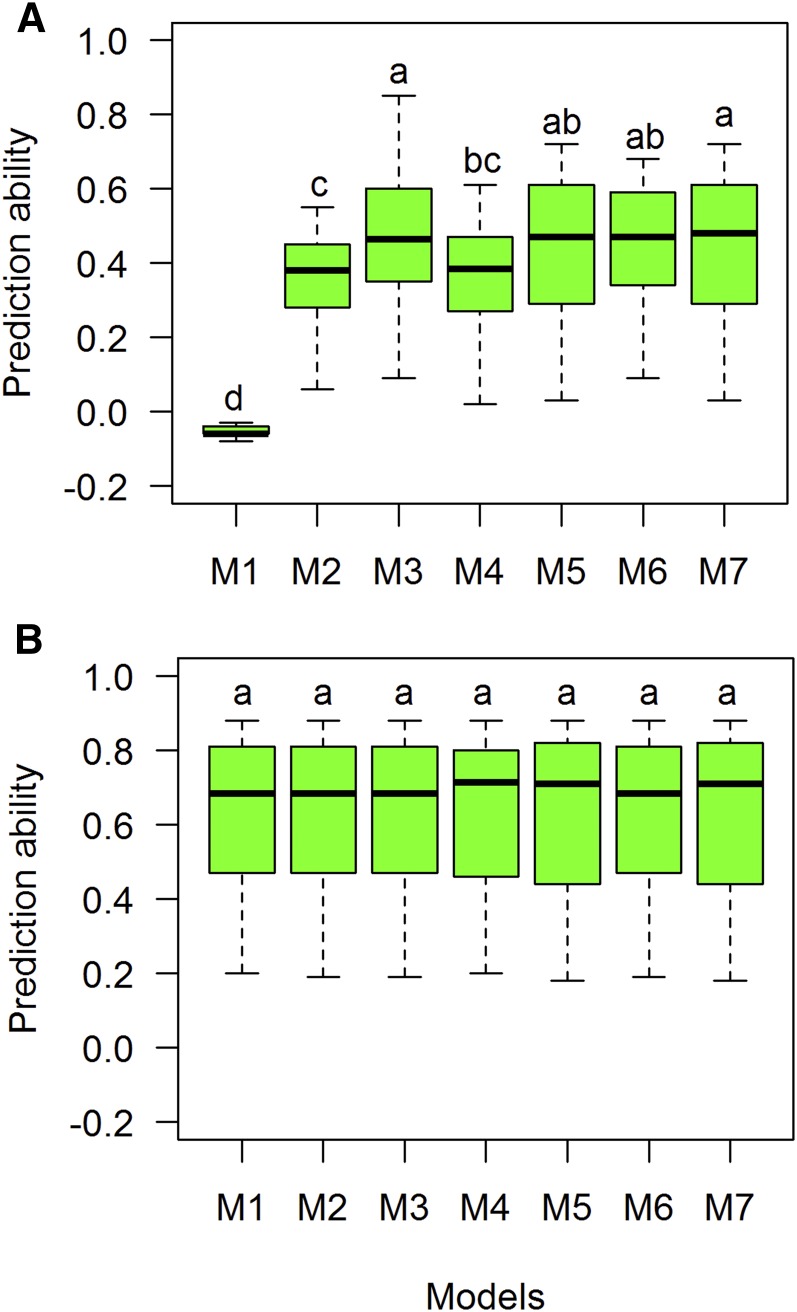
Comparison of boxplot distributions of prediction ability (correlations) for each model (M1–M7) for trait grain weight using two prediction CV scenarios: (A) CV1 and (B) CV2 for trait GW. Different letters denote significant differences among groups (*post hoc* nonparametric Tukey’s test, *P* < 0.05). Models: M1 Y = E+L+e; M2 Y= E+L+A+e; M3 Y= E+L+G+e; M4 Y= E+L+A+AE+e; M5 Y= E+L+G+GE+e; M6 Y= E+L+G+A+e; M7 Y= E+L+G+A+GE+AE+e.

Trait GW had the highest prediction values in CV1 (0.72, Iran D10) and CV2 (0.88, Iran D10) scenarios. Models 6 and 7 had eight environments with prediction values in CV1 >0.5. Thirty-six environment-model combinations had prediction values for CV1 >0.50, with M6 and M7 predicting the values for CV1 of five sites with correlation >0.6. On average, the model with the highest CV1 values was M3, followed by M7. Mex I10 and Mex D10 had the highest CV1 (0.72 for both M6 and M7) and CV2 values (0.88 for M6 and M7) (Table 4). Tukey’s test group showed models M3 and M7 as the most significant models with the highest prediction ability in the CV1 scenario (Figure 6). In the CV2 scenario, all models had the same prediction ability (0.63) (Figure 6 and Table 4).

**Table 4:** Correlations (mean ± SD) between the observed and predicted values for GW under CV1 and CV2 schemes for seven prediction models (M1–M7)

	CV1							CV2							*H*^2^
Env\Model	M1*^a^*	M2	M3	M4	M5	M6	M7	M1	M2	M3	M4	M5	M6	M7	
BGLD J10*^b^*	−0.06 ± 0.05	0.28 ± 0.02	0.39 ± 0.01	0.35 ± 0.02	0.45 ± 0.02	0.41 ± 0.02	0.46 ± 0.02	0.65 ± 0.01	0.65 ± 0.01	0.65 ± 0.01	0.69 ± 0.01	0.68 ± 0.01	0.65 ± 0.01	0.68 ± 0.01	0.69
BGLD J11	−0.06 ± 0.07	0.34 ± 0.01	0.60 ± 0.01	0.36 ± 0.01	0.43 ± 0.02	0.39 ± 0.01	0.45 ± 0.02	0.61 ± 0.01	0.61 ± 0.01	0.61 ± 0.01	0.63 ± 0.01	0.64 ± 0.01	0.61 ± 0.01	0.64 ± 0.01	0.84
India D10	−0.04 ± 0.07	0.06 ± 0.01	0.09 ± 0.01	0.02 ± 0.04	0.03 ± 0.04	0.09 ± 0.01	0.03 ± 0.04	0.20 ± 0.01	0.20 ± 0.01	0.19 ± 0.01	0.20 ± 0.01	0.18 ± 0.01	0.19 ± 0.01	0.18 ± 0.01	0
India H10	−0.06 ± 0.07	0.28 ± 0.02	0.45 ± 0.01	0.27 ± 0.02	0.29 ± 0.02	0.34 ± 0.01	0.29 ± 0.02	0.47 ± 0.01	0.47 ± 0.01	0.47 ± 0.01	0.46 ± 0.01	0.44 ± 0.02	0.47 ± 0.01	0.44 ± 0.02	0.69
India I11	−0.05 ± 0.06	0.40 ± 0.02	0.59 ± 0.01	0.41 ± 0.02	0.51 ± 0.02	0.51 ± 0.01	0.51 ± 0.02	0.78 ± 0.00	0.78 ± 0.00	0.78 ± 0.00	0.77 ± 0.01	0.77 ± 0.01	0.78 ± 0.00	0.77 ± 0.01	0.79
India K10	−0.06 ± 0.07	0.47 ± 0.02	0.51 ± 0.01	0.49 ± 0.01	0.59 ± 0.01	0.59 ± 0.01	0.59 ± 0.01	0.80 ± 0.00	0.80 ± 0.00	0.80 ± 0.00	0.79 ± 0.00	0.80 ± 0.00	0.80 ± 0.00	0.80 ± 0.00	0.77
India L11	−0.04 ± 0.06	0.21 ± 0.02	0.09 ± 0.01	0.23 ± 0.03	0.18 ± 0.03	0.19 ± 0.01	0.20 ± 0.03	0.22 ± 0.01	0.22 ± 0.01	0.22 ± 0.01	0.21 ± 0.01	0.21 ± 0.01	0.22 ± 0.01	0.21 ± 0.01	0.51
India V10	−0.04 ± 0.07	0.06 ± 0.01	0.14 ± 0.01	0.04 ± 0.04	0.04 ± 0.04	0.09 ± 0.01	0.05 ± 0.03	0.20 ± 0.01	0.19 ± 0.01	0.19 ± 0.01	0.20 ± 0.01	0.19 ± 0.01	0.19 ± 0.01	0.20 ± 0.01	0
Iran D10	−0.06 ± 0.07	0.55 ± 0.02	0.64 ± 0.01	0.61 ± 0.01	0.72 ± 0.01	0.68 ± 0.01	0.72 ± 0.01	0.84 ± 0.01	0.85 ± 0.01	0.85 ± 0.01	0.87 ± 0.00	0.88 ± 0.00	0.85 ± 0.01	0.88 ± 0.00	0.05
Mex D10	−0.04 ± 0.07	0.49 ± 0.01	0.59 ± 0.01	0.50 ± 0.02	0.64 ± 0.01	0.64 ± 0.01	0.64 ± 0.01	0.88 ± 0.00	0.88 ± 0.00	0.88 ± 0.00	0.88 ± 0.00	0.88 ± 0.00	0.88 ± 0.00	0.88 ± 0.01	0.22
Mex H10	−0.06 ± 0.07	0.45 ± 0.02	0.66 ± 0.01	0.47 ± 0.02	0.61 ± 0.01	0.59 ± 0.01	0.61 ± 0.02	0.81 ± 0.01	0.81 ± 0.01	0.81 ± 0.00	0.81 ± 0.01	0.82 ± 0.01	0.81 ± 0.00	0.82 ± 0.01	0.92
Mex HD10	−0.08 ± 0.06	0.53 ± 0.02	0.61 ± 0.01	0.57 ± 0.01	0.68 ± 0.01	0.66 ± 0.01	0.68 ± 0.01	0.87 ± 0.00	0.87 ± 0.00	0.87 ± 0.00	0.87 ± 0.00	0.88 ± 0.00	0.87 ± 0.00	0.88 ± 0.00	0.9
Mex I10	−0.05 ± 0.06	0.43 ± 0.02	0.48 ± 0.01	0.45 ± 0.01	0.64 ± 0.01	0.61 ± 0.01	0.64 ± 0.01	0.81 ± 0.01	0.81 ± 0.01	0.81 ± 0.01	0.80 ± 0.01	0.82 ± 0.01	0.81 ± 0.01	0.82 ± 0.01	0.92
Nepal B10	−0.05 ± 0.08	0.36 ± 0.02	0.45 ± 0.01	0.36 ± 0.01	0.48 ± 0.02	0.48 ± 0.01	0.49 ± 0.02	0.78 ± 0.01	0.78 ± 0.01	0.78 ± 0.01	0.77 ± 0.01	0.76 ± 0.01	0.78 ± 0.01	0.76 ± 0.01	0.95
Nepal B11	−0.06 ± 0.07	0.40 ± 0.01	0.29 ± 0.01	0.42 ± 0.01	0.46 ± 0.02	0.46 ± 0.01	0.47 ± 0.02	0.64 ± 0.01	0.64 ± 0.01	0.64 ± 0.01	0.65 ± 0.01	0.64 ± 0.01	0.64 ± 0.01	0.64 ± 0.01	0.78
Pak I11	−0.03 ± 0.04	0.18 ± 0.02	0.35 ± 0.01	0.15 ± 0.03	0.29 ± 0.03	0.29 ± 0.01	0.28 ± 0.03	0.30 ± 0.00	0.30 ± 0.00	0.30 ± 0.00	0.27 ± 0.01	0.28 ± 0.02	0.30 ± 0.00	0.28 ± 0.02	0.77
PakI10	−0.06 ± 0.07	0.40 ± 0.02	0.85 ± 0.00	0.43 ± 0.01	0.54 ± 0.01	0.52 ± 0.01	0.54 ± 0.01	0.72 ± 0.01	0.72 ± 0.01	0.72 ± 0.01	0.74 ± 0.01	0.74 ± 0.01	0.72 ± 0.01	0.74 ± 0.01	0.70
Sudan W10	−0.06 ± 0.05	0.28 ± 0.02	0.39 ± 0.01	0.35 ± 0.02	0.45 ± 0.02	0.41 ± 0.02	0.46 ± 0.02	0.65 ± 0.01	0.65 ± 0.01	0.65 ± 0.01	0.69 ± 0.01	0.68 ± 0.01	0.65 ± 0.01	0.68 ± 0.01	0.23
Average	−0.05	0.34	0.45	0.36	0.45	0.44	0.45	0.62	0.62	0.62	0.63	0.63	0.62	0.63	

Broad-sense heritability H2 of GW in each environment.

a Models: M1 Y = E+L+e; M2 Y= E+L+A+e; M3 Y= E+L+G+e; M4 Y= E+L+A+AE+e; M5 Y= E+L+G+GE+e; M6 Y= E+L+G+A+e; M7 Y= E+L+G+A+GE+AE+e.

b Names of the environments are given in Table 1.

**Figure 7:**
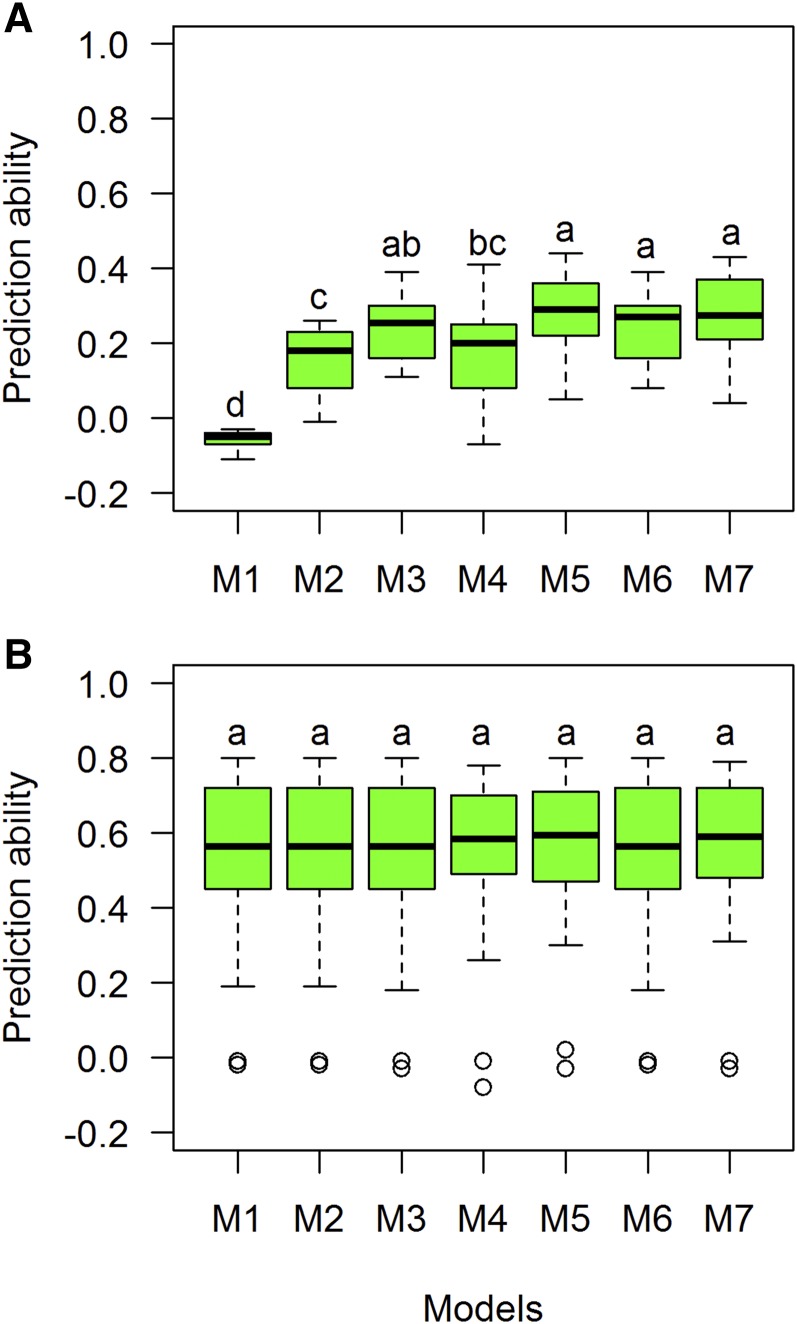
Comparison of boxplot distributions of prediction ability (correlations) for each model (M1–M7) for trait grain number using two prediction CV scenarios (A) CV1 and (B) CV2 for trait thermal time to flowering (TTF). Different letters denote significant differences among groups (*post hoc* nonparametric Tukey’s test, *P* < 0.05). Models: M1 Y = E+L+e; M2 Y= E+L+A+e; M3 Y= E+L+G+e; M4 Y= E+L+A+AE+e; M5 Y= E+L+G+GE+e; M6 Y= E+L+G+A+e; M7 Y= E+L+G+A+ GE +AE+e.

For TTF, the prediction ability in the CV1 scenario ranged from −0.11 (India L11, M1) to 0.44 (India K11, M5). A total of 29 model-site combinations had CV1 values >0.3, with M7 predicting eight sites with correlations >0.3. Mex I10 had five models predicting the sites with >0.3 for CV1, followed by Nepal. On average, M5 had the highest CV1 values (0.28) when compared with other models (Table 5). For CV2, values ranged from 0.40 (Mex H10) to 0.80 (BGLDJ10). All sites had correlation values >0.3, and 85 environment-model combinations had correlation values >0.50. Thirty-five sites (31%) had CV2 values >0.7 (Table 5). On average, correlations for M5–M7 for CV1 were 0.28, 0.24, and 0.27, respectively, and prediction ability values for M5–M7 for CV2 were 0.54, 0.52, and 0.53, respectively. Tukey’s test groups indicated that M5–M7 were the best predictive models for CV1, while for CV2 all models had the same prediction ability (Figure 7).

**Table 5:** Correlations (mean ± SD) between the observed and predicted values for TTF under CV1 and CV2 schemes for seven prediction models (M1–M7)

	CV1							CV2							*H*^2^
Env\Models	M1*^a^*	M2	M3	M4	M5	M6	M7	M1	M2	M3	M4	M5	M6	M7	
BGLD J10*^b^*	−0.03 ± 0.06	0.05 ± 0.02	0.15 ± 0.02	0.07 ± 0.05	0.27 ± 0.02	0.14 ± 0.02	0.21 ± 0.04	0.80 ± 0.01	0.80 ± 0.01	0.80 ± 0.01	0.78 ± 0.01	0.80 ± 0.01	0.80 ± 0.01	0.79 ± 0.01	0.97
BGLD J11	−0.05 ± 0.08	0.26 ± 0.04	0.33 ± 0.02	0.33 ± 0.01	0.41 ± 0.02	0.35 ± 0.02	0.41 ± 0.02	0.72 ± 0.01	0.72 ± 0.01	0.72 ± 0.01	0.68 ± 0.01	0.67 ± 0.01	0.72 ± 0.01	0.67 ± 0.01	0.84
India D10	−0.05 ± 0.05	0.09 ± 0.02	0.16 ± 0.02	−0.03 ± 0.04	0.10 ± 0.04	0.16 ± 0.01	0.07 ± 0.04	0.48 ± 0.01	0.48 ± 0.01	0.48 ± 0.01	0.51 ± 0.01	0.55 ± 0.01	0.48 ± 0.01	0.53 ± 0.01	0.53
India H10	−0.07 ± 0.06	0.09 ± 0.03	0.15 ± 0.02	0.31 ± 0.02	0.36 ± 0.02	0.16 ± 0.02	0.36 ± 0.02	0.60 ± 0.01	0.60 ± 0.01	0.60 ± 0.01	0.61 ± 0.01	0.61 ± 0.02	0.61 ± 0.01	0.62 ± 0.02	0.98
India I11	−0.07 ± 0.06	0.24 ± 0.04	0.27 ± 0.03	0.31 ± 0.02	0.35 ± 0.02	0.30 ± 0.02	0.37 ± 0.02	0.45 ± 0.01	0.45 ± 0.01	0.45 ± 0.01	0.49 ± 0.01	0.47 ± 0.01	0.45 ± 0.01	0.48 ± 0.01	0.79
India K11	−0.06 ± 0.06	0.24 ± 0.04	0.39 ± 0.02	0.25 ± 0.02	0.44 ± 0.02	0.39 ± 0.02	0.42 ± 0.02	0.64 ± 0.01	0.64 ± 0.01	0.64 ± 0.01	0.62 ± 0.01	0.64 ± 0.01	0.64 ± 0.01	0.63 ± 0.01	0.84
India L11	−0.11 ± 0.06	0.22 ± 0.08	0.21 ± 0.05	0.41 ± 0.02	0.39 ± 0.03	0.24 ± 0.05	0.43 ± 0.02	0.40 ± 0.01	0.40 ± 0.01	0.39 ± 0.01	0.39 ± 0.01	0.39 ± 0.02	0.40 ± 0.01	0.39 ± 0.02	0.98
India V10	−0.05 ± 0.07	0.15 ± 0.02	0.26 ± 0.02	0.15 ± 0.03	0.29 ± 0.02	0.26 ± 0.02	0.28 ± 0.03	0.57 ± 0.01	0.57 ± 0.01	0.57 ± 0.01	0.60 ± 0.01	0.62 ± 0.01	0.57 ± 0.01	0.61 ± 0.01	0.70
Iran D10	−0.04 ± 0.05	−0.01 ± 0.03	0.11 ± 0.02	−0.02 ± 0.04	0.05 ± 0.04	0.08 ± 0.02	0.07 ± 0.04	0.19 ± 0.01	0.19 ± 0.01	0.18 ± 0.01	0.26 ± 0.01	0.30 ± 0.01	0.18 ± 0.01	0.31 ± 0.01	0.21
Mex D10	−0.03 ± 0.06	0.08 ± 0.03	0.18 ± 0.02	0.08 ± 0.04	0.29 ± 0.03	0.18 ± 0.02	0.23 ± 0.05	0.69 ± 0.01	0.69 ± 0.01	0.69 ± 0.01	0.72 ± 0.01	0.75 ± 0.01	0.69 ± 0.01	0.74 ± 0.01	0.92
Mex H10	−0.06 ± 0.06	0.24 ± 0.03	0.25 ± 0.02	0.19 ± 0.03	0.25 ± 0.03	0.28 ± 0.02	0.24 ± 0.03	0.74 ± 0.01	0.74 ± 0.01	0.74 ± 0.01	0.75 ± 0.01	0.75 ± 0.01	0.74 ± 0.01	0.76 ± 0.01	0.83
Mex HD10	−0.04 ± 0.06	0.05 ± 0.02	0.15 ± 0.02	0.08 ± 0.04	0.28 ± 0.02	0.14 ± 0.02	0.22 ± 0.03	0.74 ± 0.01	0.74 ± 0.01	0.74 ± 0.01	0.73 ± 0.01	0.71 ± 0.01	0.74 ± 0.01	0.72 ± 0.01	0.20
Mex I10	−0.06 ± 0.06	0.23 ± 0.03	0.26 ± 0.02	0.23 ± 0.03	0.22 ± 0.03	0.29 ± 0.02	0.27 ± 0.04	0.51 ± 0.01	0.51 ± 0.01	0.50 ± 0.01	0.51 ± 0.01	0.49 ± 0.01	0.50 ± 0.01	0.49 ± 0.01	0.82
Nepal B10	−0.04 ± 0.07	0.04 ± 0.02	0.17 ± 0.01	−0.07 ± 0.04	0.09 ± 0.03	0.14 ± 0.02	0.04 ± 0.02	0.56 ± 0.01	0.56 ± 0.01	0.56 ± 0.01	0.57 ± 0.01	0.58 ± 0.01	0.56 ± 0.01	0.57 ± 0.01	0.61
Nepal B11	−0.05 ± 0.08	0.23 ± 0.03	0.32 ± 0.02	0.25 ± 0.03	0.35 ± 0.03	0.32 ± 0.02	0.34 ± 0.03	0.53 ± 0.01	0.53 ± 0.01	0.53 ± 0.01	0.55 ± 0.01	0.58 ± 0.01	0.53 ± 0.01	0.57 ± 0.01	0.77
Pak I10	−0.03 ± 0.07	0.20 ± 0.02	0.28 ± 0.02	0.11 ± 0.04	0.22 ± 0.03	0.28 ± 0.01	0.21 ± 0.03	−0.02 ± 0.01	−0.02 ± 0.01	−0.03 ± 0.01	−0.08 ± 0.02	0.02 ± 0.03	−0.02 ± 0.01	−0.03 ± 0.03	0.65
Pak I11	−0.07 ± 0.07	0.16 ± 0.02	0.30 ± 0.02	0.25 ± 0.03	0.42 ± 0.02	0.30 ± 0.02	0.40 ± 0.03	−0.01 ± 0.01	−0.01 ± 0.01	−0.01 ± 0.01	−0.01 ± 0.01	−0.03 ± 0.02	−0.01 ± 0.01	−0.01 ± 0.01	0
Sudan W10	−0.08 ± 0.06	0.20 ± 0.04	0.31 ± 0.02	0.21 ± 0.03	0.31 ± 0.03	0.32 ± 0.02	0.30 ± 0.03	0.74 ± 0.01	0.74 ± 0.01	0.75 ± 0.01	0.70 ± 0.02	0.75 ± 0.01	0.75 ± 0.01	0.74 ± 0.01	0.83
Average	−0.06	0.15	0.24	0.17	0.28	0.24	0.27	0.52	0.52	0.52	0.52	0.54	0.52	0.53	

Broad-sense heritability *H*^2^ of TTF in each environment.

a Models: M1 Y = E+L+e; M2 Y= E+L+A+e; M3 Y= E+L+G+e; M4 Y= E+L+A+AE+e; M5 Y= E+L+G+GE+e; M6 Y= E+L+G+A+e; M7 Y= E+L+G+A+GE+AE+e.

b Names of the environments are given in Table 1.

In summary, for the complex trait GY, M6 and M7 with interactions had the highest average prediction ability across environments for CV1 (0.29 and 0.31, respectively), and for CV2 (0.37 and 0.38, respectively). For the less complex trait GW, M3 and M7 showed the highest mean prediction ability for CV1 (0.45), and it was around 0.63 for all models in CV2. For grain number (GN) (which is a GY component and a complex trait), M5 and M7 gave the highest prediction ability for CV1 (0.32) and CV2 (0.43). For trait TTF, M5–M7 (0.28, 0.24, and 0.27, respectively) were the best for CV1; all models performed similarly for CV2 (0.52–0.53).

### Trends in prediction ability *vs.* heritability

**Figure B1:**
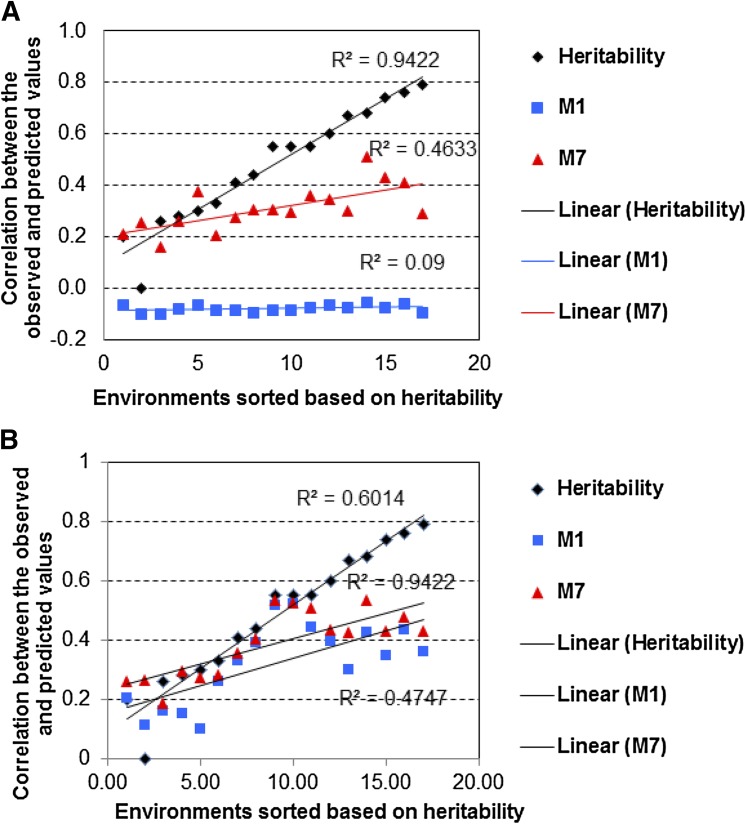
Comparison between heritability values and the correlation between observed and predicted values for the best and worst models in predicting trait GY in different environments for two cross-validation scenarios: (A) CV1 (the best and worst models were M7 and M1, respectively), and (B) CV2 (the best and worst models were M7 and M1, respectively).

**Figure B2:**
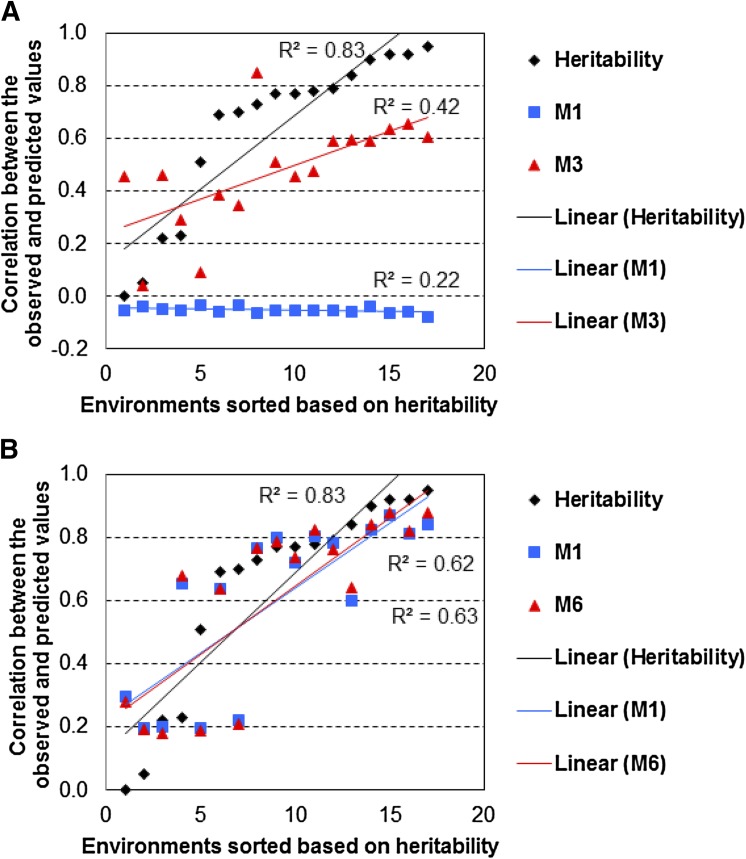
Comparison between heritability values and the correlation between observed and predicted values for the best and worst models in predicting trait GW in different environments for two cross-validation scenarios (A) CV1 (the best and worst models were M3 and M1, respectively) and (B) CV2 (the best and worst models were M6 and M1, respectively).

The best model for GY was M7 and, for CV1, it showed increasing values of environment heritability with their corresponding prediction accuracies, whereas M1 prediction ability was not related to heritability values (Figure B1-A, Appendix B). For GY in the CV2 scenario, the best and worst models had similar prediction ability, and showed an increasing trend of up to *H*^2^ = 0.50; values decreased thereafter (Figure B1-B, Appendix B). For trait GW in the CV1 scenario, a positive trend of increased prediction ability with increased *H*^2^ values was observed for the best model (M3), which had no interaction terms. The worst model (M1) did not show a response with increased *H*^2^ values (Figure B2-A, Appendix B). For the CV2 scenario, the best and worst models showed increased correlation values, and an increase in *H*^2^ values (Figure B2-B, Appendix B).

**Figure B3:**
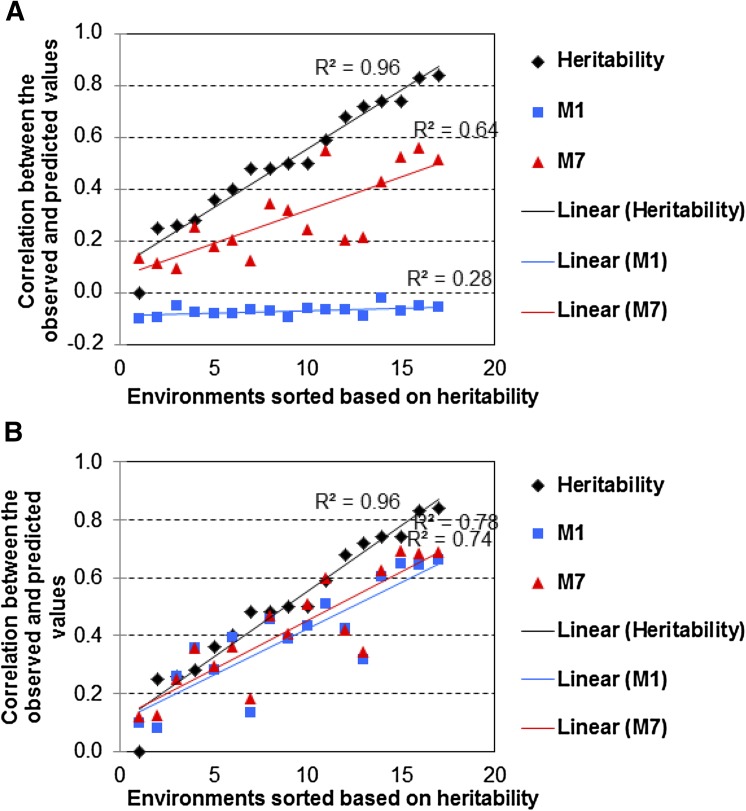
Comparison between heritability values and the correlation between observed and predicted values for the best and worst models in predicting trait GN in different environments for two cross validation scenarios: (A) CV1 (the best and worst models were M6 and M1, respectively) and (B) CV2 (the best and worst models were M6 and M1, respectively).

**Figure B4:**
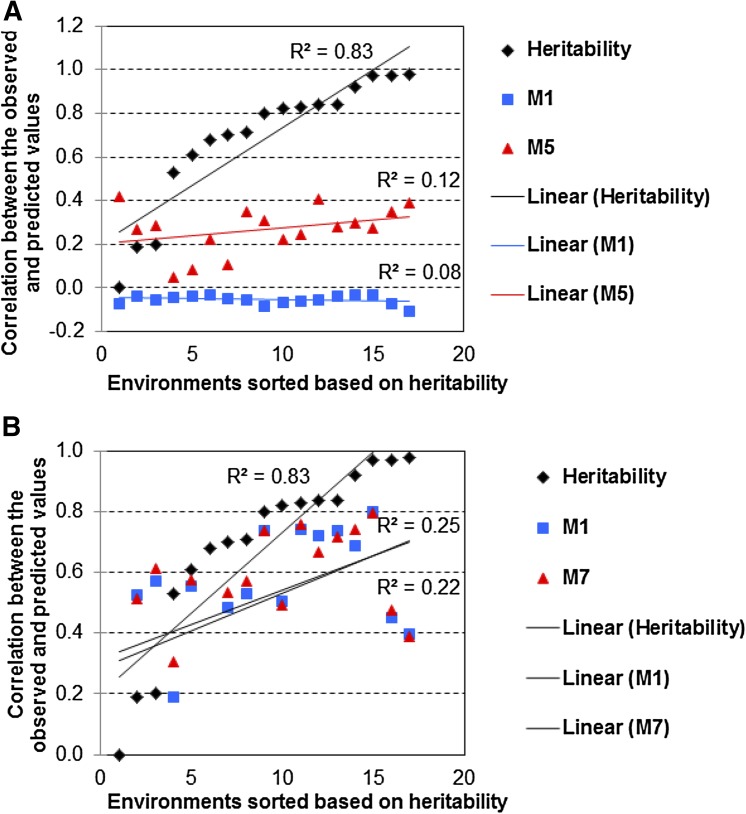
Comparison between heritability values and the correlation between observed and predicted values for the best and worst models in predicting trait TTF in different environments for two cross-validation scenarios: (A) CV1 (the best and worst models were M5 and M1, respectively) and (B) CV2 (the best and worst models were M7 and M1, respectively).

For GN, the correlations and *H*^2^ values of the best model (M7) showed a positive trend in the CV1 scenario, whereas the basic model (M1) showed no association with *H*^2^ values (Figure B3-A, Appendix B). Similar to GY, the best and basic models showed very close prediction ability and *H*^2^ values for environments in the CV2 scenario for GN (Figure B3-B, Appendix B). For TTF, the best model (M5) did not show greater prediction ability, the positive trend was lower (*R*^2^ = 0.12), and the basic model M1 showed no association with *H*^2^ values (*R*^2^ = 0.08) (Figure B4-A, Appendix B). In the CV2 scenario, the best and basic model for TTF did not show high association between prediction values and *H*^2^ estimates, with some sites with high heritability estimates showing lower prediction values (Fig. B4-B, Appendix B).

## Discussion

The WAMI panel has been extensively studied for several complex traits: adaptation to density (Sukumaran *et al.* 2015b), GY and yield components (Sukumaran *et al.* 2015a), drought stress (Edae *et al.* 2013, 2014), and earliness *per se* (Sukumaran *et al.* 2016). Since it was also phenotyped under diverse environments around the world, it is a perfect panel for testing some of the genomic and pedigree selection models. Data from these testing sites were used routinely to select lines for release as varieties, and for crossing them to generate new prebreeding lines (Reynolds and Langridge 2016). Physiological breeding is aimed at improving wheat productivity through complex physiological traits. These traits are often controlled by genes with small effects; if they can be proven to be of value in the breeding program, they are more effectively selected using genomic selection methods than using MAS.

Several models have been proposed for the genomic prediction schemes; however, it is important to test them on diverse environmental data before using them in the breeding program. Models 6 and 7 were the best models, for they had the highest average prediction ability values for the CV1 and CV2 scenarios for GY among all environments. Here, we evaluated seven models (some with the G×E term), and concluded that these models can predict GY with moderate to high levels of prediction ability, whereas less complex traits, such as GW, can be predicted without including any interaction terms in the model.

The results of this study agree with those of a recent study on Zn and Fe grain concentration in spring wheat (Velu *et al.* 2016). Models that include G×E interaction terms showed higher prediction accuracies. Also, prediction ability was generally associated with trait heritability, as in earlier reports (Muranty *et al.* 2015). The highest prediction ability was for GW, which is a high heritability trait in the WAMI panel (Sukumaran *et al.* 2015a). Another observation was that, for some environments, M3 gave high prediction ability for GW in the CV1 scenario, whereas model M2 was the best in CV2. In this study, we evaluated the correlation between genomic- and pedigree-based estimated breeding values, with phenotypic data from field trials. With a reasonable number of molecular markers, and incorporating G×E terms in the models, higher prediction ability was obtained for the “genomic” component when compared to pedigree-based prediction models (Burgueño *et al.* 2011). This was also dependent on trait heritability, as GW had higher prediction ability values even when using M3 (Muranty *et al.* 2015).

Genotypic values of lines in several environments were predicted using genomic prediction models; when compared across environments, the highest prediction ability was recorded at environments in Cd. Obregon (Mexico) for GY, GN, GW, and TTF. Relatively good climate, as well as optimal management of the Cd. Obregon site, are big factors influencing heritability of yield and prediction ability; sites with high heritability have higher prediction ability. However, our analysis also showed that there is no linear association between heritability and prediction ability values; nevertheless, prediction ability could be a function of *H*^2^ values and other parameters (Spindel *et al.* 2015). Another factor that could increase genomic prediction ability is incorporating high-dimensional environmental covariates (Jarquin *et al.* 2014; Pérez-Rodríguez *et al.* 2015). Recent studies on wheat have shown that GS selection could reshape wheat breeding because it produces higher genetic gains than conventional breeding (Bassi *et al.* 2016).

### Conclusions

Genotype × environment prediction models in genomic selection and pedigree-based selection can help accelerate breeding cycles for complex traits such as grain yield in multi-environmental trials. Traditionally, breeders have depended on phenotypic selection for generation advancement. Results of the present study show that GS is a complementary method to phenotypic selection with medium-to-high prediction ability values. Genomic prediction of GY, and other traits in spring wheat lines evaluated in a large and diverse number of international environments, indicated that sites in Mexico and India could be key sites for genomic-assisted breeding. A set of wheat lines not observed in several site-year combinations were predicted with correlations of 0.3–0.5 in Mexico and India (CV1) for models that included genomic and pedigree interaction with environments. When some of these lines were observed in some environments, this correlation increased to 0.45–0.53 (CV2).

For less complex traits, such as GW, the prediction ability of lines not observed in sets of environments increased to about 0.6 for Mexican environments (CV1). Sets of wheat lines observed in some environments, but not in others, were predicted with correlations of up to 0.8 in Mexican and India environments (CV2) for genomic-enabled prediction models including (or not) genomic and pedigree interactions with environments.

## APPENDIX A

**Table A1:** ANOVA of the 18 environments for GY, GN, GW, and TTF

Source	GY		GN		GW		TTTF	
	df	MS	df	MS	df	MS	df	MS
Env	17	1621.45***	17	10,710,471,211	17	12,355.91	16	186,946,414.
Rep (Env)	18	5.09***	18	91,729,154.79	18	138.41	17	646,553.8
Entry	293	3.89***	293	51,442,282.18	293	233.89	287	12,232,101
Env*Entry	4981	1.24***	4981	12,314,123.59	4981	15.90	4592	29,846,089
Error	5236	0.90	5236	9,058,283.004	5241	7.67	5051	8,835,189
Model R-square	0.88		0.84		0.90		0.96	
Coefficient of variation	22.7		23.65		8.41		3.33	
Mean	4.17 (ton/ha)		12,727.66 (number)		32.91 (gms)		1255.1 (°D)	
Broad-sense heritability (*H*^2^)	0.41		0.51		0.74		0.48	

df, degrees of freedom, MS mean squares, Env, environment; Rep, replication; °D, degree days.

*** significant at α = 0.001 level of significance.
